# Bilingual children acquiring Russian and German in Vienna: nonword repetition correlates with stronger but not with weaker language

**DOI:** 10.1007/s40211-023-00456-1

**Published:** 2023-02-06

**Authors:** Brigitte Eisenwort, Maksim Tilis, Carolin Schmid, Gabriela Diendorfer-Radner

**Affiliations:** 1 Department of Pediatrics and Adolescent Medicine, Medical University of Vienna, Währinger Gürtel 18–20, 1090 Vienna, Austria; 2 Department of Otorhinolaryngology, Division of Phoniatrics-Logopedics, Medical University of Vienna, Vienna, Austria; 3 Comprehensive Center of Pediatrics, Medical University of Vienna, Vienna, Austria; 4 Acoustics Research Institute, Austrian Academy of Sciences, Vienna, Austria

**Keywords:** Bilingual, Language development, Language exposure, Nonword repetition, Zweisprachig, Sprachentwicklung, Sprachexposition, Wiederholung von Pseudowörtern

## Abstract

**Purpose:**

Nonword repetition tests (NWRT) can be useful tools together with other assessment procedures for diagnosing a developmental learning disorder in bilingual children. Concerning typically developing children, however, the link between NWRT performance and language development is still unclear. The present study contributes to this discussion by investigating the link between language-dependent NWRT performance, language development, and language exposure.

**Methods:**

A total of 20 simultaneously bilingual Russian–German children, aged 4–6 years, were tested with “The Russian language proficiency test for multilingual children (SRUK)” and “Patholinguistische Diagnostik bei Sprachentwicklungsstörungen (PDSS)” as well as language-specific nonwords for Russian and German.

**Results:**

Most children scored within the mean range in SRUK. In PDSS they scored two standard deviations below the mean range in most of the subtests. NWRT in Russian significantly correlated with the NWRT in German and also positively correlated with both comprehension and production in Russian. In contrast, the German NWRT did not correlate with comprehension or with production in German. Moreover, the correlation between the German NWRT and the comprehension of grammatical structures in Russian was significant, and the correlations between the German NWRT and the two other Russian language development tests just failed to reach significance.

**Conclusion:**

High scores in both the Russian and the German NWRT offer evidence that the ability to repeat language-specific nonwords does not differ depending on language exposure. The aim to distinguish between typical and atypical language development based on NWRT can be reached only when NWRT and all possible influencing factors in typically developed children are analyzed.

## Abstract (de)

**Ziel:**

Nonword-repetition-Tests (NWRT) können gemeinsam mit anderen Screeningverfahren herangezogen werden, um eine Sprachentwicklungsstörung bei bilingual aufwachsenden Kindern festzustellen. Jedoch ist der Zusammenhang zwischen NWRT und Sprachentwicklung bei typisch entwickelten Kindern noch immer fraglich. Diese Studie liefert einen Beitrag zur Diskussion des Zusammenhangs zwischen Ergebnissen sprachabhängiger NWRT, Sprachentwicklung und Ausmaß der Sprachexposition bei bilingual aufwachsenden Kindern.

**Methode:**

Insgesamt 20 simultan bilingual Russisch-Deutsch aufwachsende Kinder zwischen 4 und 6 Jahren wurden mit dem Russischen Sprachstandtest (SRUK) und der Patholinguistischen Diagnostik bei Sprachentwicklungsstörungen (PDSS) evaluiert. Als NWRT wurden sprachspezifische Pseudowörter verwendet.

**Ergebnisse:**

Die meisten Kinder haben beim SRUK durchschnittlich abgeschnitten. Bezüglich der PDSS haben sie in den meisten Subtests zwei Standardabweichungen unter dem Durchschnittsbereich abgeschnitten. NWRT Russisch korreliert signifikant mit NWRT Deutsch als auch mit dem Sprachverständnis und der Sprachproduktion auf Russisch. Im Gegensatz dazu korreliert NWRT Deutsch weder mit dem Sprachverständnis Deutsch, noch mit der Sprachproduktion. Darüber hinaus korreliert NWRT Deutsch mit dem Verständnis grammatischer Strukturen Russisch signifikant sowie mit zwei anderen Subtests Russisch nahe der Signifikanzgrenze.

**Schlussfolgerung:**

Hohe Werte sowohl im NWRT Russisch als auch Deutsch liefern Evidenz dafür, dass die Fähigkeit, Pseudowörter zu wiederholen, unabhängig vom Ausmaß der Exposition in der betreffenden Sprache ist. Das Ziel zwischen unauffälliger und gestörter Sprachentwicklung aufgrund von NWRT zu unterscheiden, kann nur gelingen, wenn mögliche Einflussfaktoren bei typisch entwickelten Kindern analysiert werden.

## Introduction

Today a majority of children grow up multilingually, because of the many demographic changes. In Austria, German is the official language needed for the daily life and for education. Nevertheless, about 60% of children attending kindergarten in Vienna acquire another first language (L1) [1]. These children often underperform in their second language (L2) Austrian German with regard to the expectations of professionals, like teachers, psychologists, pediatricians and others. This observed lower L2 proficiency compared to monolingual children often leads them to take a developmental language disorder (DLD) into consideration because the first stages of L2 acquisition can resemble the patterns observed in impaired monolingual language development [2, 3]. There is currently a lack of language tests with norms for bilingual children, because most of the available language tests are normed for monolingual children with the aim to distinguish between typical and atypical language development. Nonword repetition tests (NWRT) are an alternative for language tests with bilingual children, because they can also be used with children who have limited exposure to a given language. In NWRT, children are asked to repeat nonwords containing one or more syllables that they have not heard before and therefore they cannot be part of their learned vocabulary. As a consequence, bilingual children with limited exposure to a language are less disadvantaged as in other language tests compared to monolingual children of the same language [4]. NWRTs directly evaluate language proficiency at the phonetic–phonological level, specifically phonological working memory, speech perception and short-term memory and indirectly give insight into phonological awareness, word learning, and overall language acquisition. Therefore, NWRTs have proven to be valuable tools for diagnosing DLD together with other tests like sentence repetition and a parent questionnaire on acquisition of the first language [5, 6]. However, there are still open questions regarding the link between NWRT performance and language development in typically developed children. While NWRT, unlike other language assessments, do not draw directly on knowledge of vocabulary and grammar, it is still influenced by language-specific knowledge, mainly phonology and phonotactics. There is evidence that children are better able to repeat nonwords, which share phonological characteristics of real words in their language. Since such knowledge relies on language exposure, bilingual children should vary in their nonword repetition performance depending on familiarity with lexical phonology of the language on which the nonwords are based upon [4, p. 126]. Up to now in the literature different results have been reported: some studies did not find effects of language exposure on bilingual children’s NWR performance (e.g., [7–9]), while other studies found a correlation between NWR performance and language exposure [10, 11]. NWR performance was shown to be significantly predicted by language proficiency, specifically by receptive and expressive vocabulary in typically developed children [12]. In a more recent study [13], the significant correlation between (passive) vocabulary and NWR proficiency was again confirmed, whereas a significant correlation between NWR proficiency and language exposure or maternal education could not be found. The authors suggest that NWR performance is an indicator of language proficiency independent of lingual and socioeconomic status.

Thus, it remains an open question whether the amount of language exposure influences not only the development of the stronger and weaker language but also performance in language-specific NWR.

## Study

### Aims

The aim of this study is to evaluate for the first time language acquisition in simultaneously bilingual children acquiring Russian and Austrian German in Vienna. Children’s language proficiency in both Russian and German is evaluated and analyzed with regard to the impact of language exposure as well as with regard to the children’s performance in language-specific NWRT.

Specifically, the following research questions are analyzed:

1. Is the higher exposure language the language in which the children’s linguistic abilities are higher, and the lower exposure language the language in which the children’s linguistic abilities are lower? Can this link be generalized to different linguistic abilities in the domains of speech comprehension and production?
2. Is proficiency in language-specific NWRT of one language correlated with linguistic ability in the other language?
3. Is proficiency in a language-specific NWRT correlated with linguistic ability in the same language?
4. Is proficiency in language-specific NWRT of one language correlated with proficiency in language-specific NWRT of the other language?
5. Are some linguistic abilities stronger correlated with NWRT proficiency than others? In order to answer this question, results of production and comprehension will be correlated separately with the results of the NWRT.

## Materials and methods

### Participants

Twenty bilingual children, acquiring Russian and German, aged from 4–6 years were selected via a Russian Facebook group. In these Facebook groups, members favor culture of their countries of origin. Inclusion criteria were that L1 Russian is the dominant language for the children at home (that is spoken to at least 60%). Both of the parents or at least one of them and additionally other caregivers and relatives speak Russian, and contact with L2 German started before the age of 25 months [14]. The children are simultaneously bilingual and started to acquire L2 at the age of 4.5 months on average. All children are exposed to Russian as their dominant language. They attend kindergarten, and score within the mean range in nonverbal cognitive ability [15]. Exclusion criteria were the presence of a hearing and/or vision impairment, a developmental disorder, a DLD (especially ICD-10, F80), and/or a chronic disease.

### Screening procedures

The children were screened in both of their languages in order to evaluate their respective language proficiencies and to subsequently correlate these results to results of the language-specific nonword repetition tests.

Regarding the evaluation of Russian, children were screened with “The Russian language proficiency test for multilingual children (SRUK)” [16]. This test is a linguistically and psycholinguistically grounded screening procedure. Bilingual children’s proficiency can be examined in the domains language production and comprehension. The test is based on preliminary norms of 167 German–Russian bilingual children between the ages of 3;0 and 6;11 years and has to be administered by a Russian native speaker. The following subtests were selected for the present study: language production (nouns and verbs), language comprehension (nouns and verbs), and comprehension of grammatical structures.

Regarding the evaluation of German, children were screened with “Patholinguistische Diagnostik bei Sprachentwicklungsstörungen (PDSS)” [17]. This procedure is linguistically and psycholinguistically grounded as well. The subtest selected were word production (nouns and verbs), word comprehension (nouns and verbs), and comprehension of syntactic structures.

NWR is influenced by phonetic and phonological development. Regarding the exclusion of children who do not have age adequate phonological abilities, the following screening procedure was performed: 5 words which were pronounced as part of the subtest active lexicon from SRUK [17] were transcribed according to International Phonetic Alphabet (IPA) from a Russian native speaker and linguist and classified according to their age-adequatedness from an experienced clinical linguist in cooperation with a speech pathologist.^1^ All children with the exception of child 3 have age adequate phonetic and phonological abilities. Child 3 had to be excluded because of a phonological disorder (ICD-10, F.80.0). The case study of this child has been published [18].

Regarding NWR in the respective languages, language-specific nonwords for Russian and German, developed by Mathieu et al. [19], were used for the present study. The nonwords were developed with regard to similarity with real words in the respective languages, concerning item length, phonotactic probability, phonological complexity, phonological similarity of the elements, and language-specific prosodic patterns [19, p. 13]. The nonwords for the respective languages were controlled for length (words with three, four, and five syllables in both languages, and in German additionally two-syllabic words), prosody (accentuated first vs accentuated last syllable) and syllable structure (consonant–vowel vs consonant–consonant–vowel).

A computer game (smartphone/tablet application) which was developed by the second author was used to elicit the nonword repetitions in a playful way inspired by the software used by Mathieu et al. [19]. In this game the children have to repeat the names of monsters.

Russian nonwords [19] are shown in Table 1.

**Table 1:** Russian nonwords (according to [19])

Syllables	Single consonants	Consonant cluster	Single consonants	Consonant cluster
3	‘p^j^a.zi.ka	gda’fu.za	‘p^j^a.mi.zu	gdu’ma.si
	‘s^j^u.vi.ta	zda’ti.va	fa’gu.ta	fka‘d^j^u.pi
	ty’ba.sa	vda’kumi	‘t^j^u.si.bi	vdu’sa.gi
4	za’ta.pi.ma	vga.pa’di.tu	zi.ka’mi.ta	kmu.vi’ga.pu
	‘vu.da.ki.za	kmu.di’sa.bu	dy.fu’di.ka	fki.bu.sa‘gu
	gi.su’da.fa	stu’ga.fa.di	fi.zu’p^j^a.di	sma.du’fi.ba
5	–	–	z^j^a.ta.vi‘pu.ga	kma.du.pa’gi.su
	–	–	ty.gu.ma’si.va	zva.ki’bu.ta.gi

German nonwords [19] are shown in Table 2.

**Table 2:** German nonwords (according to [19])

Syllables	Single consonants with final /Ə/	Consonant cluster	Single consonants	Consonant cluster with final /Ə/
*2*	’mo.gƏ	–	di’la	–
*3*	’po.da.lƏ	’pro.ka.vi	ni’to.ra	fli’po.dƏ
	’va.mi.rƏ	’tsi.ro.fa	po’za.fi	kru’ta.nƏ
	’ri.vo.nƏ	’tsu.ni.ra	zo’li.va	kla’fi.bƏ
–	Single consonants with final /Ə/	Consonant cluster with final /Ə/	Single consonants with final /Ə/	Consonant cluster with final /Ə/
*4*	fi’lo.natƏ	gli’vo.pa.nƏ	,va.ri’zo.bƏ	,blu.na’to.zƏ
	di’ka.zo.bƏ	pfu’ra.di.gƏ	,ku.mi’da.fƏ	,fla.mo’di.zƏ
	va’ni.zu.tƏ	pro’zi.na.tƏ	,mu.za’ti.lƏ	,kro.fi’na.tƏ
–	–	–	Single consonants with final /Ə/	Consonant cluster with final /Ə/
*5*	–	–	,tu.va.lo’mi.bƏ	,blu.ri.zo’makƏ

### Analysis

All children (except child 3 who was excluded from the analysis) could be classified as having typical language development. As the aim of the NWRT was to measure the ability to repeat syllables, NWR abilities of the children were evaluated with tolerance. This means that phonetic errors and typical phonological processes are not counted. Consequently, the obtained raw scores per child could vary from 0–28 (see also Mathieau et al. [19]). For a descriptive analysis of the language abilities in the respective languages, and thus, in order to answer research question 1, the results of the language screenings are shown in percentage ranks (for the PDSS), and in raw scores (for SRUK). Results of SRUK were analyzed by transforming the raw scores into four categories according to Gargarina et al. [16]: within normal range, above normal range, conspicuous, and very conspicuous based on provisional norms. This was in order to perform a descriptive analysis of the screening results in relation to the biographic data. Then, a statistical analysis of the relation between the NWRT and language development in both languages was performed.

For statistical analyses, and thus, in order to answer research questions 2–5, one-sided correlation tests were performed in R [20]. The tests were one-sided, because the hypotheses postulate a positive and not a negative relation between the nonword repetition tests and language development at the diverse linguistic levels. Correlation tests were performed between the NWRT in Russian and the NWRT in German, and a set of three correlation analyses was performed between each of the NWRT and all three subtests of language development in one language, i.e., the comprehension of verbs and nouns, the comprehension of grammar, and the production of verbs and nouns. As all variables are interval scaled, Pearson’s product moment correlation coefficient was calculated for variables that were normally distributed. Tests of normality were performed with the Shapiro–Wilk test. Correlation tests involving variables that were not normally distributed were performed by calculating Spearman’s rho (ρ). Moreover, *p*-values of all correlation tests were Bonferroni corrected in order to account for type 1 errors, because of the multiple tests in which the variables of the NWRT were involved. In this case, the significance level of *p* > 0.05 was divided by 3, i.e., the number of correlation tests performed within each set of correlation analysis, leading to a corrected significance level of 0.05/3 = 0.017.

## Results

Sociodemographic parameters show that 84.2% of mothers and 57.9% of fathers have a university degree and 42.1% of the households has an income of more than 50,000 € (Table 3).

**Table 3:** Sociodemographic parameters concerning socioeconomic status

Parental education				
*Highest level of education*	Lower grade	Sixth grade	University degree	No information
*Mother (%)*	–	15.8	84.2	–
*Father (%)*	15.8	21.8	57.9	5.3
Yearly household income				
*Income (€)*	< 12,000	12,000–28,000	28,000–50,000	> 50,000
*Number of households (%)*	21.1	5.3	31.6	42.1

**Fig. 1:**
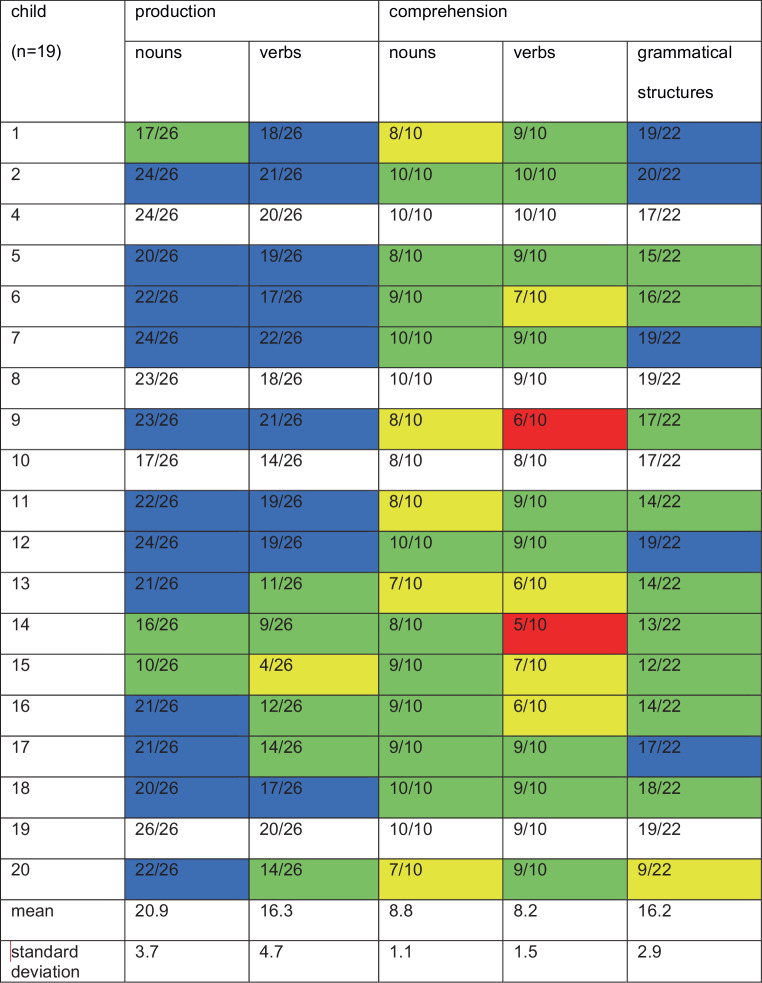
Language proficiency in Russian (highly exposed language) in speech production and comprehension. Results of the “The Russian language proficiency test for multilingual children (SRUK)” in raw scores (attained raw score/maximally achievable scores), and classified in levels of proficiency according to [16]: *green shaded* average range, *blue shaded* above average, *yellow shaded* conspicuous, i.e., about 1 standard deviation (SD) below average, but norm values are not yet available, *red shaded* very conspicuous, i.e., 2 SD below average, but norm values are not yet available, and *white fields* indicate that norm values are not available for the age group of the child

**Fig. 2:**
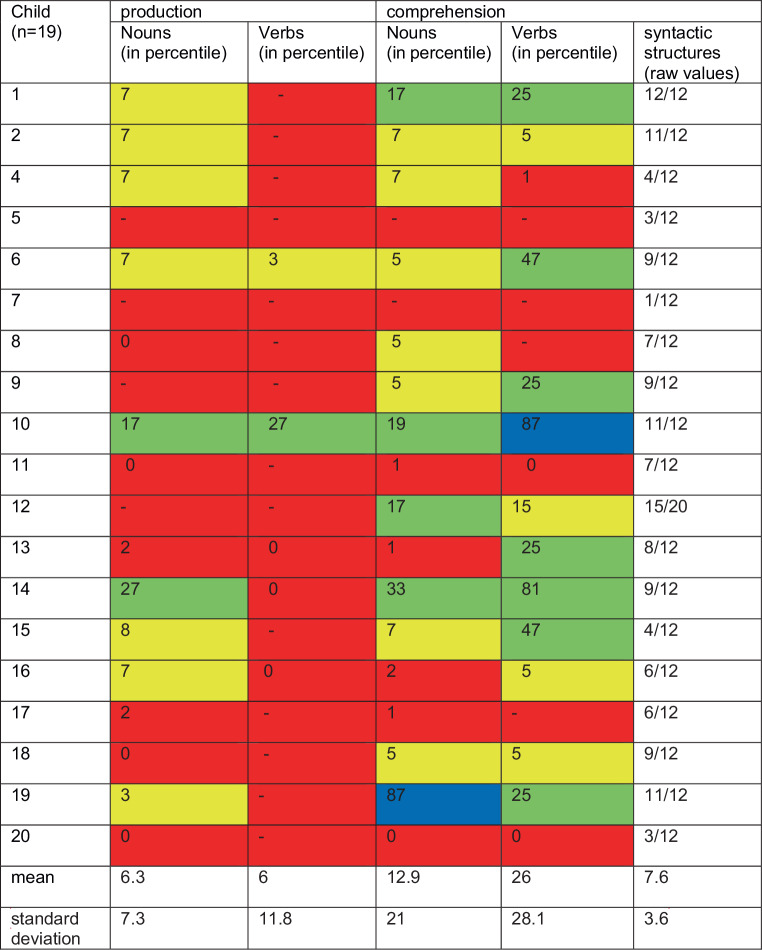
Language proficiency in German (lower exposed language) in speech production and comprehension. Results of the “Patholinguistische Diagnostik bei Sprachentwicklungsstörungen (PDSS)” in percentiles (except the results for the comprehension of syntactic structures, which are shown in raw scores: attained raw score/maximally achievable scores). Colors indicate levels of proficiency: *green shaded* average range, *blue shaded* > 1 standard deviation (SD) above average, *yellow shaded *> 1 SD below average, *red shaded* > 2 SD below average

A descriptive analysis of the children’s language proficiency in Russian and German shows that all children (except child 3 who was excluded from further analyses, see above) could be classified as having a typical language development (Figs. 1 and 2).

Results of the Russian language tests showed that most of the bilingual children scored within the mean range in all subtests. Only in the subtest comprehension of verbs did two children score two standard deviations below average. In the other subtests, none of the children received such low scores. In their L2 German, in contrast, most of the children scored two standard deviations below the mean range in most of the subtests. In German, only one child attained results in the average range or higher in all subtests. In their high exposure language Russian, the variation of test results between children was small, whereas in the low exposure language German the variation was large.

### Correlations

Shapiro–Wilk normality tests showed that all variables but two were normally distributed. The two variables production of Russian verbs and nouns, and the comprehension of Russian grammar did not show a normal distribution. Thus, correlations involving one of these two not normally distributed variables were calculated with Spearman’s ρ, whereas the other correlations were analyzed with Pearson’s product moment correlation coefficient.

Results showed that the NWRT in Russian significantly correlates with the NWRT in German, r (17) = 0.8, *p* < 0.001. The higher children’s scores were in the Russian NWRT, the higher their scores were also in the German NWRT.

The NWRT in Russian was shown to be positively correlated both with language comprehension (verbs and nouns, grammatical structures) in Russian, and also with language production (verbs, nouns) in Russian. The correlation between the NWRT in Russian and the comprehension of grammatical structures in Russian was shown to be significant (Spearman’s ρ = 0.68, *p* < 0.001). Thus, the higher the children’s scores were in NWR, the higher their scores were also in the comprehension of grammatical structures in Russian. This also holds for the comprehension of nouns and verbs: The higher the children’s scores were in the Russian NWRT, the higher their scores were also in the comprehension of nouns and verbs in Russian. However, the correlation between the NWRT in Russian and the comprehension of nouns and verbs in Russian just failed significance, r (17) = 0.46, *p* = 0.02. The correlation between the NWRT in Russian and the production of nouns and verbs in Russian was shown to be significant (Spearman’s ρ = 0.58, *p* < 0.001). This means that the higher children’s scores in the Russian NWRT were, the higher their scores were in the production of nouns and verbs in Russian. In contrast, the NWRT in German did not correlate with language comprehension in German (with nouns and verbs: r (17) = 0.2, *p* = 0.2, and with grammatical structures: r (17) = 0.26, *p* = 0.14), nor with language production of nouns and verbs in German, r (17) = 0.26, *p* = 0.14.

In addition, the positive correlation between the NWRT in Russian and the NWRT in German (see above) was investigated in more detail, insofar as the strength of the direct correlation between the German NWRT and the language development tests in Russian was analyzed. Results showed that the correlation between the NWRT in German and the comprehension of grammatical structures in Russian was significant, Spearman’s ρ = 0.54, *p* = 0.009, but the correlations between the NWRT in German and the two other Russian language development tests just failed to reach significance (for the correlation with language comprehension of nouns and verbs, r (17) = 0.33, *p* = 0.09, and for the correlation with the production of verbs and nouns, Spearman’s ρ = 0.35, *p* = 0.07).

## Discussion

In the present study, language proficiency and its correlation with NWRT was tested in 20 bilingual children who were between 4 and 6 years of age and who simultaneously acquired Russian and Austrian German. All of the bilingual children live in families representing a higher social class of Russian migrants in Vienna. Their stronger language Russian is spoken from one or even both of the parents at home more often than German. The results of this study confirm that the language to which bilingual children are most often exposed evolves into their stronger language in terms of proficiency of language comprehension and production. Results of the Russian language tests showed that most of the bilingual children scored within the mean range. Only in the subtest comprehension of verbs were the scores of two children very conspicuous. In the other subtests, none of the children received such low scores. In their L2 German, in contrast, most of the children scored two standard deviations below the mean range in most of the subtests. The participants live in families who are members of a Russian Facebook group and favor Russian language and culture. Many studies show that input plays an important role for language proficiency since it activates the innate structure of language in children (e.g., [21]). The children of this study have more language exposure in Russian, and develop Russian as their stronger language and German as their weaker language. Furthermore, concerning NWRT, the results show that Russian NWRT and German NWRT correlate, suggesting that the children’s ability to repeat syllables they do not usually hear and that reflect the phonology of the respective language is connected in the two languages of the children. NWRT in German does not reflect the lower input in German as compared to the higher input in Russian. The bilingual children in this study had high scores in both the Russian and the German NWRT. The data, thus, offers evidence that the ability to repeat language-specific nonwords does not differ depending on language exposure (classifying Russian as the high exposure language, and German as the low exposure language). The results for language exposure and NWRT in L2 German to some extent support results previously reported in the literature. Thordadottier and Brandeker [7], for example, found that NWR was less affected by previous language exposure than vocabulary measures in simultaneously bilingual French–English children. Similar results were reported by Thordadottier and Juliusdottier [8], who found that their L2 Icelandic bilingual children achieved high scores in NWRT and low scores in a measure of language. Brandeker and Thordadottier [9] also concluded that NWRT and vocabulary measures are differently associated to language exposure insofar that vocabulary acquisition is associated in a moderate to strong manner to language exposure, whereas the relationship of exposure with NWRT is weak or nonsignificant.

The results of this study also suggest that language exposure influences which of the languages of typically developed simultaneously bilingual children evolves into their stronger or the weaker language, but that language exposure does not influence the performance in NWRT. However, concerning the correlation between language proficiency tests and NWRT, the results of this study suggest a differentiation according to language exposure. Whereas NWRT correlates with language comprehension and production in Russian, i.e., in the language to which the children were most exposed, there is no correlation between NWRT and language comprehension and production (nouns and verbs) in German, namely in the language to which the children were less exposed. Moreover, in the children’s stronger language Russian, language proficiency is linked to NWRT in both L1 Russian and L2 German, whereas language proficiency in their weaker language German is not linked to NWRT in either of the two languages.

An explanation of this discrepancy in the correlation between language proficiency and NWRT in the results could be that Russian phonology and phonotactics are more complex than German phonology and phonotactics (see, for example, [22]), suggesting that acquisition of the complex structures of Russian phonology encloses acquisition of simpler structures of German phonology. Makarova and Therekova [23] who studied the acquisition of Russian phonology in children in a migration context state that these children show similar characteristics concerning the development of their phonetic inventory and phonological processes as compared to their monolingual peers, because they had a high level of Russian language exposure in their families. The children of this study also have a high level of Russian language exposure in migration context and the authors therefore assume that their phonetic inventory and phonological processes are comparable with their monolingual Russian peers. So acquisition of these complex phonological structures in L1 Russian could enclose simpler structures in L2 German. As Ulbrich and Wiese [22] state, Russian shows a more extended patterning of consonant clusters than German. Thus, it differs from German for example in the complexity of possible sound combinations in the syllable coda, in that Russian allows more consonant sequences and more frequent violation of sonority requirements compared to German. We suggest that, at least in the case of the Russian–German bilingual children of this study, language exposure only indirectly influences performance in NWRT, insofar as NWRT in both languages is linked to language development in the stronger language Russian, to which the children are more exposed to and which shows more phonotactic complexity compared to German. Further research desiderata that build on the present study are necessary: studies investigating the correlation between NWRT and language proficiency in L1 and L2 in German–Russian bilingual children whose stronger language is German instead of Russian, studies regarding acquisition of expressive and receptive phonology in bilingual children acquiring Russian and German simultaneously as well as studies investigating the correlation between NWRT and language proficiency in bilingual and typically developing children acquiring other combinations of languages. The aim to distinguish between typical and atypical language development based on NWRT can be reached only when NWRT and all possible influencing factors like, for example, the role of language exposure in typically developed children are analyzed.

## Limitations

The most important limitation of our study is the size of the group which characterizes it as a pilot study. It focuses only on one pair of languages Russian and German. Even though the findings show that NWRT is not affected by language exposure, the authors did not differentiate between cumulative and current exposure and used a cumulative measure of exposure [9]. Further limitations are that language skills in both languages are measured with a screening method instead of standardized tests, and that only a small number of participants are included.

## Conclusions

Many bilingual children are suspected to have a developmental language disorder (DLD) because of their lower proficiency their second language (L2). Language evaluation in both languages is complicated by many facts like lack of normed language tests and different amount of exposure in the languages. The nonword repetition test (NWRT) is influenced by language-specific knowledge but does not draw directly on knowledge of vocabulary and grammar. Therefore, we recommend to use language-specific NWRT in bilingual children who are suspected to have DLD, but to interpret test results with caution. There are still many open questions regarding the influence of language exposure and phonological structure of different languages on NWRT performance.

## Footnotes

1 Transcription according to International Phonetic Alphabet (IPA) was done by Dmitry Tsoy. Dagmar Formann, speech pathologist, cooperated in screening of five words according to their age-adequateness.
