# Erratum to: Bilingual children acquiring Russian and German in Vienna: nonword repetition correlates with stronger but not with weaker language

**DOI:** 10.1007/s40211-023-00463-2

**Published:** 2023-03-22

**Authors:** Brigitte Eisenwort, Maksim Tilis, Carolin Schmid, Gabriela Diendorfer-Radner

**Affiliations:** 1 Department of Pediatrics and Adolescent Medicine, Medical University of Vienna, Währinger Gürtel 18–20, 1090 Vienna, Austria; 2 Department of Otorhinolaryngology, Division of Phoniatrics-Logopedics, Medical University of Vienna, Vienna, Austria; 3 Comprehensive Center of Pediatrics, Medical University of Vienna, Vienna, Austria; 4 Acoustics Research Institute, Austrian Academy of Sciences, Vienna, Austria

**Erratum to:**

**Neuropsychiatr 2023**

10.1007/s40211-023-00456-1

Due to a mistake, the special characters in Table 2 were not displayed in the PDF.

**Table 2:** German nonwords (according to [19])

Syllables	Single consonants with final /Ə/	Consonant cluster	Single consonants	Consonant cluster with final /Ə/
*2*	’mo.gƏ	–	di’la	–
*3*	’po.da.lƏ	’pro.ka.vi	ni’to.ra	fli’po.dƏ
	’va.mi.rƏ	’tsi.ro.fa	po’za.fi	kru’ta.nƏ
	’ri.vo.nƏ	’tsu.ni.ra	zo’li.va	kla’fi.bƏ
–	Single consonants with final /Ə/	Consonant cluster with final /Ə/	Single consonants with final /Ə/	Consonant cluster with final /Ə/
*4*	fi’lo.natƏ	gli’vo.pa.nƏ	,va.ri’zo.bƏ	,blu.na’to.zƏ
	di’ka.zo.bƏ	pfu’ra.di.gƏ	,ku.mi’da.fƏ	,fla.mo’di.zƏ
	va’ni.zu.tƏ	pro’zi.na.tƏ	,mu.za’ti.lƏ	,kro.fi’na.tƏ
–	–	–	Single consonants with final /Ə/	Consonant cluster with final /Ə/
*5*	–	–	,tu.va.lo’mi.bƏ	,blu.ri.zo’makƏ

The original article has been corrected.

